# Investigation of Voids in the Apical Plug of MTA Using Cone-Beam Computed Tomography, Digital Radiography, and Analog Radiography 

**DOI:** 10.30476/dentjods.2025.104334.2520

**Published:** 2026-03-01

**Authors:** Mahsa Eskandarinezhad, Sedighe Razi, Tirdad Pirzadeh, Sabete Bagheri Sabzevar, Leyla Nazari, Salehe Akhondian

**Affiliations:** 1 Dept. of Endodontics, Faculty of Dentistry, Tabriz University of Medical Sciences, Tabriz, Iran.; 2 Dept. of Oral and Maxillofacial Radiology, Faculty of Dentistry, Tabriz University of Medical Sciences, Tabriz, Iran.; 3 Dentist, Tabriz, Iran.; 4 Dept. of Endodontics, Faculty of Dentistry, Mashhad University of Medical Sciences, Mashhad, Iran.; 5 Dept. of Endodontics, Faculty of Dentistry, Ardabil University of Medical Sciences, Ardabil, Iran.; 6 Student Research Committee, School of Dentistry, Mashhad University of Medical Sciences, Mashhad, Iran.

**Keywords:** X-Ray Films, Cone Beam Computed Tomography, Dental Digital Radiography

## Abstract

**Background:**

Achieving a satisfactory apical seal in root canals with open apices can be challenging, with the risk of filling material extrusion into the apex. Timely detection of voids in root canal filling material is crucial to prevent complications.

**Purpose:**

This study aimed to compare the accuracy of cone-beam computed tomography (CBCT), digital radiography, and conventional radiography in detecting voids in mineral trioxide aggregate (MTA) apical plugs.

**Materials and Method:**

In this in vitro study, thirty-two extracted upper maxillary incisors underwent decoronation, root canal instrumentation, and simulation of an apexification model for open apex teeth. The samples were then divided into two groups (n=16) based on void sizes including group A in which MTA was compacted manually as an apical plug, and group B in which MTA apical plug was made with simulated voids using an 0.4-mm diameter needle. CBCT, digital, and analog radiography scans were performed on all samples. The images were assessed by an endodontist and a radiologist to identify the presence of voids. The accuracy of the three methods in detecting voids in MTA apical plugs was evaluated using Chi-square analysis.

**Results:**

CBCT (43.8%) showed superior detection of small voids (group A) compared to analog (37.5%) and digital (18.7%) radiography methods, with analog radiography being more effective than digital radiography (*p*= 0.037). For large void detection (group B), digital radiography (94%) outperformed the other methods, while analog (81.3%) and CBCT (81.3%) techniques exhibited similar diagnostic abilities (*p*= 0.034). The significance level was set at *p*< 0.05.

**Conclusion:**

CBCT imaging is more effective than analog radiography for detecting small voids, and both methods outperform digital radiography, likely due to CBCT's three-dimensional imaging capabilities. In diagnosing large voids, digital radiography showed higher accuracy than the other techniques, while CBCT and analog radiography had similar diagnostic abilities. Variations in image processing and radiation doses used might account for the differences between these techniques.

## Introduction

Teeth with open apices or enlarged apical constrictions can present a challenge during endodontic treatment, making it difficult to achieve a satisfactory apical seal [ 1 ]. Problems may arise during root canal filling, including the risk of material extrusion into periapical tissues and the inability to fill all dimensions without an apical stop [ 2 ]. A potential solution for cases with over instrumented apices is using mineral trioxide aggregate (MTA) to create a mineralized tissue barrier, similar to apexification treatment [ 2 ]. 

MTA has been shown to possess favorable physical, chemical, and biological properties and is commonly used for procedures like perforation repair, retro filling, pulp capping, and apexification [ 3 - 4 ]. It offers advantages over calcium hydroxide, such as shorter treatment periods, reduced risk of tooth fracture, and fewer dental office visits [ 5 ]. Many experts consider MTA the preferred material for creating an apical plug in cases of necrotic pulp and wide-open apices due to its excellent sealing ability [ 6-7 ]. However, it is important to note that MTA can be costly and challenging to apply in the apical area, with a risk of inadvertent extrusion into periapical tissues that could damage tissues and complicate the repair process [ 8 ]. 

The primary objective of successful root canal therapy is the accurate filling of the prepared root canal system, but complications can arise during the procedure. One such issue is the presence of voids in the apical and coronal parts of the root canal filling material, which can create pathways for leakage and contribute to treatment failure [ 9 ]. Insufficient obturation has been documented to cause voids inside the canal filling and at the interface between the filling and dentin, allowing bacterial migration in the coronal-apical direction or vice versa, which may result in reinfection or chronic apical periodontitis [ 10 ]. 

Clinical radiographs are commonly used to assess the quality of root canal fillings due to their non-invasiveness and ethical considerations. Both conventional and digital intraoral radiographs are commonly used to evaluate endodontic treatment, but these two-dimensional images have limitations in providing detailed information [ 11 ]. Voids in root canal fillings may be challenging to detect in the buccolingual dimension, especially in canals with oval or ribbon shapes that extend widely in that direction [ 12 ]. 

Intraoral sensors require lower radiation doses compared to conventional films, reducing overall absorbed doses. However, digital systems like photostimulable phosphor (PSP) plates are susceptible to bending and scratching, resulting in permanent image artifacts. Furthermore, maintaining infection control with digital receptors can pose challenges [ 13 ]. 

Cone-beam computed tomography (CBCT) imaging is a non-invasive method for examining dentoalveolar structures, offering high resolution and two-sided radiation. Its primary benefit lies in the three-dimensional nature of the images, avoiding issues like superimposition and geometric distortion [ 12 ]. 

Kositbowamchai . [ 14 ] compared direct digital images with conventional radiographs in detecting simulated root canal voids. Their findings indicated that the diagnostic capabilities of digital and conventional images for detecting voids in root canal fillings did not differ significantly. Similarly, Huybrechts . [ 15 ] compared the effectiveness of intraoral analog, digital, and CBCT images in identifying root canal-filling voids. Their research concluded that digital intraoral techniques outperformed analog and CBCT images in detecting small voids, although voids larger than 300 micrometers were non-significantly detectable with all imaging methods. The size of voids can impact the success of root canal treatment, with different radiographic techniques capable of detecting varying void sizes [ 15 ]. 

This study compared the detection of voids in MTA apical plugs using different radiographic imaging methods, including intraoral analog, digital, and CBCT images, focusing on small and simulated void sizes. 

## Materials and Method

Thirty-two extracted maxillary permanent incisors were used in the study and stored in normal saline. The crowns were cut to 12 mm each using a high-speed bur with water spray. Radiopaque markers were placed 3 mm from the apex of each root using aluminum foil to identify the void formation sites.

The root canals were instrumented using Gates-Glidden drills from #1 to #5 via the crown-down technique to create the apexification model. Gates-Glidden drill #5 reached a depth of 3mm, drill #4 reached 5 mm, drill #3 reached 7mm, and drill #2 was inserted 9 mm into the canal to establish an open apex state. Drill #1 was used 1mm beyond the foramen to pass once without resistance, followed by a rotary instrument (RaCe, FKG Dentaire, La-Chaux-de-Fonds, Switzerland) entering the root canal from the apical foramen to the entire crown length. Root canal irrigation consisted of 5mL of 2.5% sodium hypochlorite (NaOCl) and 5mL of 17% EDTA to remove debris and smear layer, followed by another NaOCl rinse.

The teeth were then placed in floral foam to simulate periapical tissues and randomly divided into two groups based on void sizes. Due to the lack of a completely similar study, a pilot study was used to determine the number of samples, and ultimately 32 samples (2 groups of 16) were considered for the study.

In group A, MTA (Angelus, Brazil) was prepared according to the manufacturer’s instructions, placed in the root canal using an MTA carrier, packed with appropriate-size pluggers to a depth of 3 mm from the root ends, and followed by inserting a paper cone soaked in a phosphate-buffered saline solution into the root canals. Once removed from the floral foam, cotton soaked in a phosphate-buffered saline solution was placed at the apical ends of the roots. In group B, after MTA placement, the samples were positioned at a 3mm distance from the apex using a 0.4mm-diameter syringe needle between the MTA and the root canal wall, packed as in group A. The syringe head was maintained in place until the MTA set and then removed. The samples were stored in 100% humidity at 37°C for one week; with periapical tissues reconstructed using floral foam.

CBCT images were captured using a Newtom VGI device (Verona QR, Italy) at 10 ms and 110 kVp concerning axial sections. Data from the CBCT scans was input into NNT view version 2.17 software for analysis on a 19-inch LCD monitor (Philips) with a resolution of 1024*1208 and 32 bits.

For digital radiography, each root was exposed using digital radiography equipment (Kodak, France) with a parallel technique. The data was stored on a 19-inch monitor with a resolution of 32 bits and examined at 2× magnification (Kodak, France).

Conventional film images were taken using #2 E-speed periapical films (10 mA radiation, 60 kVp) from the same company as the digital equipment.

Two independent observers, an endodontist and an oral radiologist, evaluated all the images under the same conditions using a magnifying glass at 2× magnification at a distance of 50-100cm. Observers with a kappa value of ≥0.80 for void detection were chosen for the study, noting the presence or absence of voids in each image. Also, the study was blinded and the observers were una-ware of the presence or absence of voids in the samples.

Statistical analysis was conducted using SPSS 17 and descriptive statistics (frequency percentages). The chi-squared test was used to compare groups, with a significance level of *p*< 0.05.

## Results

**Figure 1:**
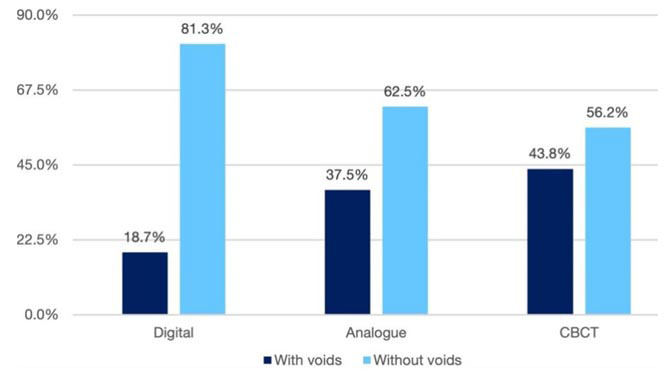
Comparison of the presence of voids in the mineral trioxide aggregate (MTA) apical plug with three imaging methods in group A (*Cone-beam computed tomography (CBCT))

Based on the study’s significance level (*p*< 0.05), the chi-squared test indicated a significant difference between the three techniques. In particular, the CBCT method demonstrated superior detection of small voids compared to the analog and digital methods, with analog also outperforming digital (*p*= 0.037) (Figure 1).

**Figure 2:**
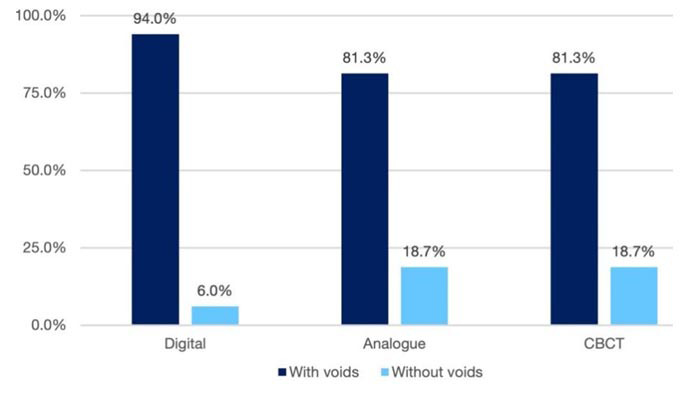
Comparison of the presence of voids in the mineral trioxide aggregate (MTA) apical plug with three imaging methods in group B (*Cone-beam computed tomography (CBCT))

Moreover, concerning large void detection, digital radiography was superior to the other methods, while analog radiography and CBCT exhibited similar diagnostic capabilities (*p*= 0.034) (Figure 2). 

## Discussion

The presence of voids in root canal fillings can impact treatment outcomes, making their detection crucial yet challenging. Radiographs taken after treatment are critical for evaluating root canal filling quality and detecting voids [ 15 ]. This study compared the diagnostic accuracy of digital and analog radiographs with CBCT images in void detection.

Significantly, the results revealed discrepancies in void presence within the MTA apical plug across the three methods. CBCT imaging proved more effective in identifying small voids compared to analog and digital radiography, with analog also being superior to digital. CBCT offers higher image resolution than conventional radiography, allowing better visualization of small structures like voids in root canals. It also provides superior contrast, improving the differentiation between materials like gutta-percha and surrounding tissues, which helps detect small voids. Additionally, CBCT's wide field of view enables a 3D view of the entire root canal system, making it easier to detect voids, particularly in areas not well captured by 2D radiographs [ 15 ]. The superior detection of small voids by CBCT suggests that this imaging technique could be crucial for improving the prognosis of MTA filling treatments, particularly in cases where voids in the apical third may lead to less favorable outcomes [ 16 ]. Identifying such voids early could potentially allow clinicians to intervene before complications arise, thus improving long-term treatment success.

On the other hand, digital radiography effectively detected large voids, while analog and CBCT showed comparable performance. The higher effectiveness of digital radiography in detecting larger voids emphasizes its importance in evaluating root canal fillings, especially in cases where retreatment is required. The ability to detect larger voids accurately may lead to more informed clinical decisions, ultimately improving patient outcomes [ 17 ].

The current study deliberately controlled the size and location of voids in radiographs to simplify image interpretation. Voids in the apical or middle third of the root canal are associated with a less favorable prognosis compared to those in the coronal third or no voids at all [ 18 ]. This study specifically focused on void detection in the apical third of root canal fillings.

The size of voids plays a critical role in the success of root canal treatment, with different radiographic techniques varying in their ability to detect void sizes. In this study, simulated voids were set at 0.4mm, while previous research by Da Silva . [ 19 ] reported an average void length of 0.9±0.6mm per root canal. Huybrechts . [ 15 ] investigated the detection of voids >300µm with all the three imaging techniques. Digital intraoral methods outperformed intraoral analog and CBCT images for small void detection. Notably, the simulated void size in our study was larger than in previous research. An in vitro study by Aghdasi . [ 11 ], focusing on maxillary incisor teeth, demonstrated that digital images could identify artificial voids with a 0.2mm diameter more effectively than conventional radiographs, consistent with our findings.

A study by Kositbowornchai . [ 14 ] revealed no significant difference between digital and conventional imaging in diagnosing voids in root canals filled with gutta-percha, contrasting with the findings of the present study. This difference in results may stem from the disparities in filling materials, as MTA was used in our study. Wonkyung .’s research [ 3 ] in 2016 supported this notion by demonstrating a higher void percentage with MTA as an orthograde obturation material compared to gutta-percha. Their study, focusing on mesial and distal canals of extracted human mandibular molars, highlighted the influence of both the apical plug material and root morphology on void formation [ 3 ].

Another study by Møller . [ 17 ] on the diagnostic difference between CBCT and digital receptors to identify voids in gutta-percha root canal filling concluded that CBCT cannot be recommended for assessing the quality of root canal fillings. In this study, CBCT axial sections exhibited a higher sensitivity, but at the same time, overdiagnosis of voids was seen, possibly due to artifacts from the gutta-percha root canal fillings. In contrast, given the findings of Schäfer .’s experimental study [ 20 ], which showed no significant variation in void detection in curved canals filled with gutta-percha between analogue and digital radiography, adopting non-destructive, three-dimensional scanning techniques could prove advantageous.

Improving radiographic quality can enhance endodontic evaluations in clinical settings, although previous studies have yielded conflicting results with different methodologies [ 14 - 15 ]. The current study explored the impact of different imaging techniques (CBCT, digital, and analog) on void sizes in MTA apexification. While numerous investigations have assessed root canal filling quality with gutta-percha or apexification with calcium hydroxide using various imaging techniques, research on MTA is limited [ 2 , 11 ]. Future studies should aim for more comprehensive evaluations across all tooth types, including those with complex canals and different void types.

This study has certain limitations that should be considered when interpreting the results. Firstly, the use of extracted teeth in an in vitro setting may not fully replicate in vivo conditions, where factors such as surrounding tissues, physiological movement, and fluid dynamics could influence void detection. Additionally, while efforts were made to standardize void simulation, potential biases in the creation and placement of voids may have impacted the findings. Furthermore, the study focused exclusively on MTA as the apexification material, which may limit the generalizability of the results to other materials commonly used in endodontic treatments. Lastly, the controlled environment of this study does not account for variations in clinician experience, image interpretation skills, or patient-related factors that could affect diagnostic accuracy in a real-world clinical setting [ 21 ]. Future studies should aim to address these limitations by incorporating in vivo conditions, assessing different filling materials, and evaluating the influence of operator experience on diagnostic outcomes. Future research could focus on optimizing digital radiography techniques to improve their sensitivity in detecting smaller voids, potentially bridging the gap between the performance of digital and CBCT imaging. Additionally, exploring other imaging modalities could provide further insights into their utility in endodontic diagnosis and treatment planning. The integration of these findings into clinical practice could ultimately lead to more precise and effective treatment planning, improving patient care and outcomes in endodontics.

## Conclusion

In conclusion, void size and imaging technique emerged as determining factors. Digital receptors were more effective in identifying voids >0.4mm than analog and slightly outperformed CBCT techniques. For detecting smaller voids, CBCT images proved superior to both intraoral analog and digital techniques. The clinical implications of these findings are significant. CBCT's ability to detect small voids in the apical third of root canal fillings may play a key role in improving the prognosis of treatments by allowing earlier intervention. On the other hand, the superior performance of digital radiography in detecting larger voids can help clinicians make more informed decisions about treatment options, such as retreatment or monitoring, to ensure the long-term success of endodontic procedures [ 19 ].

## References

[1] Mente J, Hage N, Pfefferle T, Koch MJ, Dreyhaupt J, Staehle HJ, et al ( 2009). Mineral trioxide aggregate apical plugs in teeth with open apical foramina: a retrospective analysis of treatment outcome. J Endod.

[2] Souza RA, Silva-Sousa YT, Colombo S, Lago M, Duarte MA, Pécora JD ( 2012). Healing of a tooth with an overinstrumented apex, extensive transportation and periapical lesion using a 5 mm calcium hydroxide apical plug: an 8-year follow-up report. Braz Dent J.

[3] Jho W, Park JW, Kim E, Song M, Seo DG, Yang DK, et al ( 2016). Comparison of root canal filling quality by mineral trioxide aggregate and gutta percha cones/AH plus sealer. Dent Mater J.

[4] Harinkhere C, Patni PM, Jain P, Raghuwanshi S, Pandey SH, Bilaiya S ( 2024). Comparison of the sealing ability amongst orthograde apical plugs of mineral trioxide aggregate plus, mineral trioxide aggregate repair HP, and Biodentine after root resection: a bacterial leakage study. Odontology.

[5] Holland R, Filho JA, de Souza V, Nery MJ, Bernabé PF, Junior ED ( 2001). Mineral trioxide aggregate repair of lateral root perforations. J Endod.

[6] Güneş B, Aydinbelge HA ( 2012). Mineral trioxide aggregate apical plug method for the treatment of nonvital immature permanent maxillary incisors: Three case reports. J Conserv Dent.

[7] Cervino G, Laino L, D'Amico C, Russo D, Nucci L, Amoroso G, et al ( 2020). Mineral Trioxide Aggregate Applications in Endodontics: A Review. Eur J Dent.

[8] Bogen G, Kuttler S ( 2009). Mineral trioxide aggregate obturation: a review and case series. J Endod.

[9] Wu MK, Dummer PM, Wesselink PR ( 2006). Consequences of and strategies to deal with residual post-treatment root canal infection. Int Endod J.

[10] Zhang P, Yuan K, Jin Q, Zhao F, Huang Z ( 2021). Presence of voids after three obturation techniques in band‐shaped isthmuses: a micro‐computed tomography study. BMC Oral Health.

[11] Aghdasi MM, Asnaashari M, Aliari A, Fahimipour F, Soheilifar S ( 2011). Conventional versus digital radiographs in detecting artificial voids in root canal filling material. Iran Endod J.

[12] Venkatesh E, Elluru SV ( 2017). Cone beam computed tomography: basics and applications in dentistry. J Istanb Univ Fac Dent.

[13] Brennan J ( 2002). An introduction to digital radiography in dentistry. J Orthod.

[14] Kositbowornchai S, Hanwachirapong D, Somsopon R, Pirmsinthavee S, Sooksuntisakoonchai N ( 2006). Ex vivo comparison of digital images with conventional radiographs for detection of simulated voids in root canal filling material. Int Endod J.

[15] Huybrechts B, Bud M, Bergmans L, Lambrechts P, Jacobs R ( 2009). Void detection in root fillings using intraoral analogue, intraoral digital and cone beam CT images. Int Endod J.

[16] Lee AH, Cheung GS, Wong MC ( 2012). Long-term outcome of primary non-surgical root canal treatment. Clin Oral Investig.

[17] Møller L, Wenzel A, Wegge-Larsen AM, Ding M, Væth M, Hirsch E, et al ( 2013). Comparison of images from digital intraoral receptors and cone beam computed tomography scanning for detection of voids in root canal fillings: an in vitro study using micro-computed tomography as validation. Oral Surg Oral Med Oral Pathol Oral Radiol.

[18] Naitoh M, Yuasa H, Toyama M, Shiojima M, Nakamura M, Ushida M, et al ( 1998). Observer agreement in the detection of proximal caries with direct digital intraoral radiography. Oral Surg Oral Med Oral Pathol Oral Radiol Endod.

[19] Da Silva D, Endal U, Reynaud A, Portenier I, Orstavik D, Haapasalo M ( 2002). A comparative study of lateral condensation, heat-softened gutta-percha, and a modified master cone heat-softened back-filling technique. Int Endod J.

[20] Schäfer E, Nelius B, Bürklein S ( 2012). A comparative evaluation of gutta-percha filled areas in curved root canals obturated with different techniques. Clin Oral Investig.

[21] Elendu C, Amaechi DC, Okatta AU, Amaechi EC, Elendu TC, Ezeh CP, et al ( 2024). The impact of simulation-based training in medical education: A review. Medicine (Baltimore).

